# The potential diagnostic accuracy of urine formaldehyde levels in Alzheimer’s disease: A systematic review and meta-analysis

**DOI:** 10.3389/fnagi.2022.1057059

**Published:** 2022-12-13

**Authors:** Fan Chen, Na Wang, Xinyan Tian, Yan Qin, Juan Su, Rongqiao He, Xiaping He

**Affiliations:** ^1^School of Basic Medical Sciences, Dali University, Dali, Yunnan, China; ^2^State Key Laboratory of Brain and Cognitive Science, Institute of Biophysics, Chinese Academy of Sciences, Beijing, China; ^3^Key Laboratory of Mental Health, Institute of Psychology, Chinese Academy of Sciences, Beijing, China

**Keywords:** Alzheimer’s disease, meta-analysis, formaldehyde, biomarkers, diagnosis

## Abstract

**Background:**

Formaldehyde (FA), a toxic aldehyde, has been shown to be associated with a variety of cognitive disorders, including Alzheimer’s disease (AD). There is increasing evidence that FA levels are significantly increased in AD patients and may be involved in the pathological process of AD. The aim of this study was to assess the potential diagnostic value of urine FA levels in AD using meta-analysis techniques.

**Methods:**

Original reports of morning urine FA levels in AD patients and healthy controls (HCs) were included in the meta-analysis. Standardized mean differences (SMD) were calculated using a random-effects model, heterogeneity was explored using methodological, age, sex difference and sensitivity analyses, and receiver operating characteristic (ROC) curves were constructed to assess the diagnostic value of urine FA levels in AD.

**Results:**

A total of 12 studies were included, and the urine FA levels of 874 AD patients and 577 HCs were reviewed. Compared with those in HCs, the FA levels were significantly increased in AD patients. The heterogeneity of the results did not affect their robustness, and results of the area under the curve (AUC) suggested that urine FA levels had good potential diagnostic value.

**Conclusion:**

Urine FA levels are involved in AD disease progression and are likely to be useful as a potential biomarker for clinical auxiliary diagnosis. However, further studies are needed to validate the results of this study.

## Introduction

Alzheimer’s disease, a common age-related dementia with the senile plaques and the neurofibrillary tangles (NFTs) as the main histopathological hallmarks (Ballard et al., 2011; Scheltens et al., 2016), accounts for the greatest number of vascular dementias, has increased in incidence, and 80 million patients are expected to be diagnosed with AD worldwide by 2030 (Gauthier et al., 2021). However, thus far, the etiology of AD remains unexplained, and the mechanisms underlying the etiology of AD, including external environment, age, and familial inheritance, are extremely complex (Fyfe, 2017). It is concerning that there have been no new effective advances in novel agents for the treatment of AD. Therefore, the key to the treatment of AD, such as cancer, is early diagnosis, intervention and delay the process of AD in the early stage of the disease.

However, it has been reported that more than 75% of AD patients worldwide are currently undiagnosed due to the availability of economic resources among AD patients as well as the difficulty of early clinical diagnosis (Gauthier et al., 2021). Early diagnosis of AD currently relies on a combination of cognitive tests and imaging. Although emerging biomarkers related to AD have been studied (Karaboğa and Sezgintürk, 2022; Martinez and Peplow, 2022; Song et al., 2022), the severity of AD necessitates the identification of biomarkers which are more convenient, cost-effective, and highly sensitive. Blood and urine are readily available for testing, and the development of additional urine tests should be focused on due to its availability for non-invasive collection.

Since the typical pathological feature of AD is amyloid β (Aβ) protein deposition and Tau protein hyperphosphorylation, current studies on humoral diagnostic markers for AD mainly focus on the levels of Aβ and Tau protein-related markers in cerebrospinal fluid (CSF) and blood (Bjerke and Engelborghs, 2018). For Aβ, combined detection of Aβ42 and Aβ40 can be used to monitor AD progression and prognosis (Lewczuk et al., 2015; Ren et al., 2022). For Tau protein, pTau181 levels in plasma can reflect the dynamic disease progression of AD (Moscoso et al., 2021). pTau217 also possesses good specificity early in the course of AD (Ren et al., 2022). In China, lumbar puncture CSF examination can be performed in AD patients only when they are hospitalized (Ren et al., 2022), which places a part of the limitation on clinical application. Although these markers in CSF and blood have a high diagnostic accuracy, they cannot be widely used in clinical practice due to their invasiveness. In addition, amyloid protein showed significant cohort differences between positive and negative individuals, suggesting that Aβ may lack robustness as a biomarker itself (West et al., 2021). Similarly, the accuracy of pathological Tau as a biomarker remains to be validated with a large number of clinical applications (Ren et al., 2022). In that case, markers in urine have emerged as potential clinical diagnostic criteria due to its availability for non-invasive collection (Zhang et al., 2014). In a recent clinical study, urine AD7c-NTP showed good diagnostic sensitivity (89.3%) and specificity (84.7%) (Ma et al., 2016). Therefore, other components in urine also deserve further attention and exploration.

Formaldehyde (FA), the main raw material and byproduct of industrial and commercial production, is readily available in the external environment and in the cells of vertebrates, including humans (Zhao et al., 2021). Recent studies have shown that FA-induced neurotoxicity plays an important role in the pathogenesis of AD and cognitive impairment. Elevated endogenous FA has already been detected in the brains of AD patients (Lu et al., 2013). Increasing FA levels bring about memory decline and learning dysfunction while inducing damage and demyelination in the hippocampus (Kilburn et al., 1987; Perna et al., 2001; Gurel et al., 2005; Tang et al., 2012). Interestingly, abnormally elevated FA levels were found in the urine, blood, CSF, and postmortem hippocampus of AD patients (Yang et al., 2014). These injuries to the nervous system make FA a risk factor for AD, especially in the early stages of AD pathogenesis (Song and Wang, 2010; Rizak et al., 2014).

In humans, endogenous FA is mainly excreted in the urine. Since there are few proteins in urine that react with FA (Tong et al., 2011), we speculated that urine FA may be a useful non-invasive biomarker for the diagnosis of AD. Some studies have illustrated the association between urine FA and AD by demonstrating that elevated urine FA levels are inversely correlated with cognitive competence in AD patients (Wei-shan et al., 2010; Tong et al., 2011, 2014, 2016; Zhi-Hui et al., 2011; Li et al., 2016, 2017; Jihui et al., 2017; Yi et al., 2017; Ai et al., 2019; Kun et al., 2019; Shou-zi et al., 2019). Therefore, based on the close relationship between FA and AD pathogenesis, we performed the current meta-analysis to assess the accuracy of the reported association between urine FA and the etiology of AD and to further determine whether an increase in FA levels can be used as a biomarker for the clinical diagnosis of AD. Our findings suggest an association between urine FA levels and AD that can be used for risk assessment or as a clinical aid in the diagnosis of AD.

## Materials and methods

### Protocol registration

We conducted a meta-analysis based on observational epidemiological studies (MOOSE; Stroup et al., 2000). The current study was registered in the International Prospective Register of Systematic Reviews (PROSPERO) under registration number CRD42021283692.

### Retrieve strategies and data extraction

We conducted a systematic computerized literature search of the PubMed, Web of Science, Cochrane Library, Wan Fang (WF), China Science and Technology Journal, and China National Knowledge Infrastructure (CNKI) databases. The literature search was performed using the following Medical Subject Heading (MeSH) terms and keywords: “Alzheimer’s disease” OR “Alzheimer Dementia(s)” OR “Senile Dementia” OR “Alzheimer Type Dementia” OR “Alzheimer Type Senile Dementia” OR “Senile Dementia, Alzheimer Type” OR “Presenile Dementia” OR “Familial Alzheimer Disease” OR “Early Onset Alzheimer Disease” OR “Presenile Alzheimer Dementia” AND “Formaldehyde” OR “Oxomethane” OR “Methanal” OR “Formol” OR “Formalin.” The search included human publications without any language restrictions, and the data were collected separately by the two reviewers (FC and NW) in November 2021. Disagreements were resolved through discussion with the corresponding author (XH). We manually retrieved the references listed in the retrieved review articles to broaden the literature search. We extracted relevant data from each study, including the author name, publication year, number of AD patients and healthy controls (HCs), urine FA levels in AD patients and HCs, and methods used to detect urine FA levels.

### Study inclusion criteria and exclusion

The inclusion criteria of the retrieved literature were as follows: (1) case-control study; (2) AD patients were diagnosed using criteria from the Diagnostic and Statistical Manual of Mental Disorders (DSM-IV) or the American Psychiatric Association (APA) or the American Academy of Neurology, Language Disorders, and Stroke Senile Dementia and Related Disorders (NINCDS-ADRDA); (3) controls were healthy volunteers without age or sex differences, without cognitive impairment and abnormal psychiatric symptoms; (4) FA levels were measured from fasted urine samples collected in the morning; and (5) no language barriers precluding study participation.

The exclusion criteria of the retrieved literature were as follows: (1) FA levels obtained from cell lines, animals, brain tissue, CSF, and peripheral blood; (2) controlled studies; (3) missing information or incomplete publications; (4) reviews, case reports, and conference papers; and (5) systematic reviews or meta-analyses.

The final decision regarding inclusion was decided by consensus among the reviewers.

### Quality assessment

Two researchers independently used the Newcastle-Ottawa scale (NOS) (Lo et al., 2014) to assess the quality of the included articles. The use of NOS is supported by the Cochrane Collaboration (Higgins and Green, 2011). A study had a maximum score of two stars for comparability. Notably, scores equal to or higher than six stars were considered to indicate high quality of the study, and the number of stars was directly related to the quality of studies.

### Statistical analysis

The significance of the standardized mean difference (SMD) and its corresponding 95% confidence interval (CI) were used to compare differences in the FA levels between AD patients and HCs (Higgins et al., 2003). When the included studies listed SMD values, we used the recommended formula for estimation. The heterogeneity between studies was later assessed using Cochran’s chi-square test (CHI^2^), with *I*^2^ values of 25, 50, and 75% corresponding with low, medium, and high heterogeneity, respectively (Higgins et al., 2003). Random effects meta-analysis models were used for highly heterogeneous results and these were visualized in the form of forest plots. In the sensitivity analysis, studies were individually removed to examine the effect of each individual study on the pooled risk estimates (Pan et al., 2018). In addition, funnel plots were used to assess for publication bias when the number of eligible studies included in the analysis was greater than 10 (DerSimonian and Laird, 1986). After constructing the receiver operating characteristic (ROC) curve, the diagnostic performance of urine FA levels was obtained by calculating the area under the curve (AUC). The optimal cutoff point and Youden’s index were determined by the maximum sensitivity and specificity.

Data analysis was based on Review Manager 5.3 and STATA 12.0.

The results were visualized using R (Version 3.6.3).

All hypotheses were tested, and *p* values of less than 0.05 were considered significant.

## Results

### Literature retrieve results and study characteristics

**FIGURE 1:**
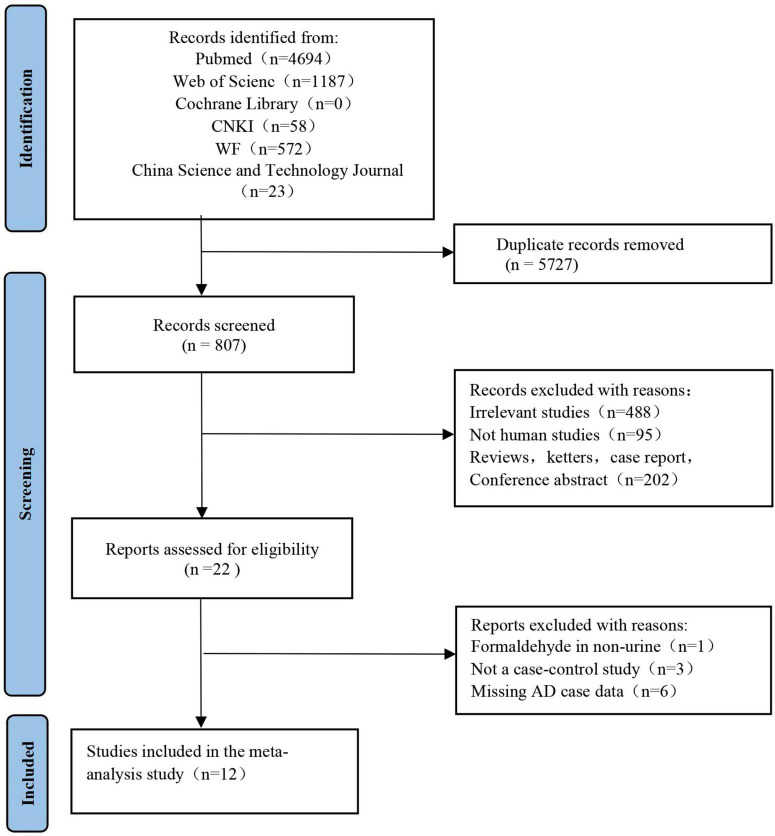
Study selection process for meta-analysis using preferred reporting items for systematic reviews and meta-analyses (PRISMA) guidelines. The figure shows the articles retrieved from the database and the criteria for selection and exclusion.

Two independent reviewers (FC and NW) reviewed the retrieved articles by checking the titles, abstracts, and full texts in the order given. A flow chart of the literature search in this meta-analysis is provided in Figure 1. The search results yielded 4,694 articles from PubMed, 1,187 from Web of Science, 1,198 from CNKI, 572 from the WF database, and 23 from the China Science and Technology Journal database. We found that 5,727 articles in the above databases were duplicates. Of the remaining 807 papers, 488 irrelevant research papers, 95 papers on non-human clinical experiments, and 202 reviews and conference abstracts were excluded. Finally, in this meta-analysis, we included a total of 12 eligible articles related to urine FA levels in AD patients.

Table 1 shows the quality evaluation results of the included studies in this meta-analysis. The NOS scores ranged from 6 to 9. Table 2 shows the details of the 12 included studies.

**TABLE 1:** Quality assessment according to the nine-star Newcastle-Ottawa scale (NOS).

ID	Authors (years)	Selection				Comparability		Exposure			Overall quality
					
		Adequate definition of the cases	Representativeness of the cases	Selection of controls	Definition of controls	Comparability of cases and controls on the basis of the design or analysis		Ascertainment of exposure	Same method of ascertainment for cases and controls	Non-response rate	
1	Kun et al. (2019)	★	★	✩	★	★	★	★	★	★	8
2	Li et al. (2017)	★	★	★	★	★	✩	★	★	✩	7
3	Zhi-Hui et al. (2011)	★	✩	★	★	★	★	★	★	✩	7
4	Tong et al. (2016)	★	★	★	★	★	★	★	★	★	9
5	Tong et al. (2011)	★	✩	✩	★	★	★	★	★	★	7
6	Wei-shan et al. (2010)	✩	★	★	★	★	✩	★	★	✩	6
7	Yi et al. (2017)	★	✩	✩	★	★	★	★	★	★	7
8	Li et al. (2016)	★	★	★	★	★	✩	★	★	★	8
9	Jihui et al. (2017)	★	★	✩	★	★	★	★	★	★	8
10	Ai et al. (2019)	★	✩	✩	★	★	★	★	★	★	7
11	Shou-zi et al. (2019)	★	✩	★	★	★	✩	★	★	★	7
12	Tong et al. (2014)	★	✩	✩	★	★	✩	★	★	★	6

Solid star represents one score, and hollow star represents zero score.

**TABLE 2:** Characteristics of each study included in the meta-analysis.

ID	Authors	Years	N (Male)		Age (SD)		FA level (SD)		Units	Method	References
			AD	HC	AD	HC	AD	HC			
1	Kun et al.	2019	113 (41)	70 (23)	78.06 (6.32)	78.02 (6.90)	15.10 (3.21)	10.47 (3.60)	μmol/L	HPLC	(Kun et al., 2019)
2	Ma-li et al.	2017	82 (50)	74 (50)	74.5 (1.3)	73.41 (1.07)	6.89 (0.62)	4.73 (0.36)	μg/L	Fluo-HPLC (FA/URC)	(Li et al., 2017)
3	Zhi-Hui et al.	2011	90 (44)	30 (13)	76.2 (6.84)	78.34 (7.15)	0.1572 (0.1451)	0.0409 (0.0303)	mmol/L	Fluo-HPLC	(Zhi-Hui et al., 2011)
4	Tong et al.	2016	42 (21)	42 (21)	77.312 (3.148)	75.523 (3.252)	0.0556 (0.050)	0.0296 (0.0136)	mmol/L	Fluo-HPLC	(Tong et al., 2016)
5	Tong et al.	2011	141 (64)	38 (18)	73 (NA)	67.373 (0.661)	0.266 (0.385)	0.083 (0.006)	mmol/L	Fluo-HPLC	(Tong et al., 2011)
6	Wei-shan et al.	2010	30 (14)	30 (13)	78.16 (8.74)	79.03 (7.62)	0.26 (0.22)	0.04 (0.02)	mmol/L	Fluo-HPLC	(Wei-shan et al., 2010)
7	Yi et al.	2017	45 (34)	10 (5)	78.504 (NA)	78.4 (NA)	45.37 (13.77)	15.3 (5.4)	μmol/L	Electrophoretic Determination	(Yi et al., 2017)
8	Li et al.	2016	62 (22)	69 (29)	81.05 (6.36)	67.33 (6.19)	1.71 (0.86)	1.11 (0.37)	μmol/L	HPLC (FA/URC)	(Li et al., 2016)
9	Jihui et al.	2017	52 (23)	53 (24)	74.67 (9.70)	73.42 (8.57)	13.27 (4.16)	10.76 (4.47)	μmol/L	HPLC	(Jihui et al., 2017)
10	Ai et al.	2019	30 (12)	52 (21)	80.02 (2.36)	77.01 (3.45)	56.67 (10.27)	24.43 (2.47)	μmol/L	Fluo-HPLC	(Ai et al., 2019)
11	Shou-zi et al.	2019	44 (18)	54 (23)	78.84 (1.33)	77.4 (1.67)	6.04 (0.77)	4.99 (0.42)	NA	HPLC (FA/URC)	(Shou-zi et al., 2019)
12	Tong et al.	2014	143 (66)	55 (34)	79.4 (6.12)	79.5 (4.2)	0.042 (0.029)	0.029 (0.015)	mmol/L	Fluo-HPLC	(Tong et al., 2014)

AD, Alzheimer’s disease; HC, healthy controls; N, number; FA, formaldehyde; SD, standard deviation; HPLC, high performance liquid chromatography; URC, urinary creatinine ratio.

### Studies on urine formaldehyde levels between Alzheimer’s disease patients and healthy controls

**FIGURE 2:**
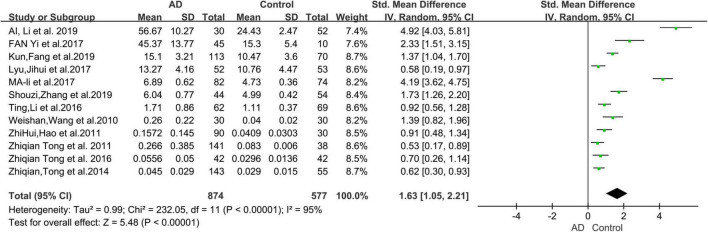
Forest plot of a random effects meta-analysis of urine FA levels in AD patients and HCs. Diamonds represent combined effect estimates. The size of the green box is proportional to the weight assigned to each study, with horizontal lines representing 95% confidence interval (CI). AD, Alzheimer’s disease; FA, formaldehyde; SD, standard deviation.

The 12 selected studies included a total of 1,451 participants (874 AD patients and 577 HCs), and we compared the urine FA levels between the AD patients and HC subjects in these samples. The random effects meta-analysis showed that urine FA levels were significantly higher in the AD patients than that in the HCs (SMD = 1.63; 95% CI: 1.05–2.21; *p* < 0.00001; Figure 2). However, substantial heterogeneity was found in the meta-analysis studies (*I*^2^ = 95%; *p* < 0.00001; Figure 2).

### Subgroup analyses

**FIGURE 3:**
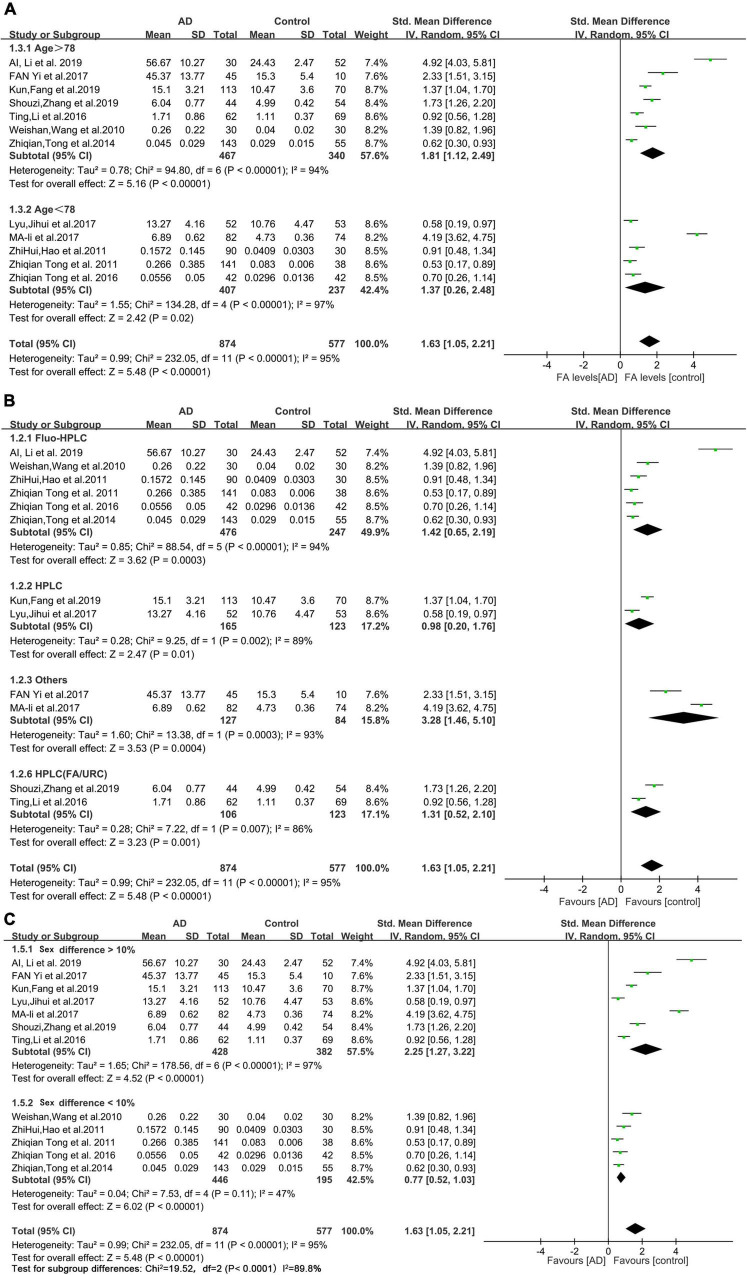
Forest plot of subgroup analyses. **(A)** Results of a random-effects meta-analysis of urine FA levels in AD patients (Age > 78 or age < 78) and HCs (*p* < 0.00001). **(B)** Random effects meta-analysis results of different detection methods for urine FA levels (*p* < 0.00001). **(C)** Random effects meta-analysis results of urine FA levels in AD patients (sex difference > 10% or sex difference < 10%) and HCs (*p* = 0.004). Diamonds represent combined effect estimates. The size of the green box is proportional to the weight assigned to each study, with horizontal lines representing 95% CI. AD, Alzheimer’s disease; SD, standard deviation.

The results of the subgroup analyses are shown in Figure 3. The mean age of the AD patients included in this study (78 years) was used as the cutoff value for the subgroup analysis. The urine FA levels were considerably higher in the AD patients older than 78 years than that in the HCs (SMD = 1.81, 95% CI: 1.12–2.49, *p* < 0.00001; Figure 3A). When the urine FA levels were measured using the Fluo-HPLC assay and high performance liquid chromatography (HPLC) assay, similar conclusions were drawn; that is, the urine FA levels were significantly higher in AD patients than in HCs. There was no significant difference between the two basic test methods (Fluo-HPLC: SMD = 1.42, 95% CI: 0.65–2.19, *p* < 0.00001; HPLC: SMD = 0.98, 95% CI: 0.20–1.76, *p* = 0.002; Figure 3B). Heterogeneity remained when using sex differences for subgroup analyses (*I*^2^ = 87.8%, *p* = 0.004; Figure 3C). There was heterogeneity among the different studies, indicating that age, measurement method and sex were not important sources of heterogeneity. The results of the subgroup analyses were not subject to publication bias because the number of studies in each comparison was less than 10.

### Sensitivity analysis and publication bias

**FIGURE 4:**
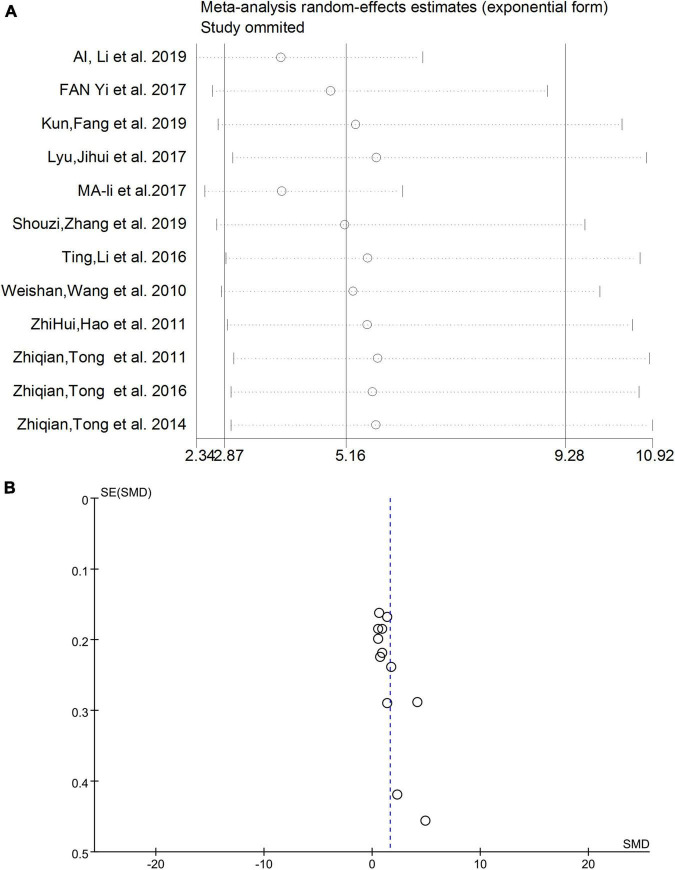
**(A)** Sensitivity analysis excluding individual studies. **(B)** Funnel plot results of meta-analysis. SMD, standardized mean difference.

After eliminating studies individually, the sensitivity analysis showed that no study had a substantial impact on the results (Figure 4A). Therefore, heterogeneity did not affect the robustness of individual studies in this meta-analysis. The funnel plot results are shown in Figure 4B, and no obvious publication bias was observed.

### Correlation between urine formaldehyde levels and degrees of Alzheimer’s disease

**FIGURE 5:**
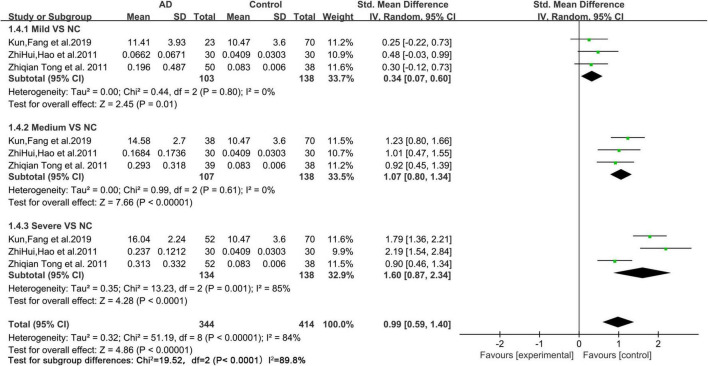
Forest plot of a random effects meta-analysis of urine FA levels and degrees of AD (mild, medium, and severe) (*p* < 0.0001). Diamonds represent combined effect estimates. The size of the green box is proportional to the weight assigned to each study, with horizontal lines representing 95% CI. SD, standard deviation; AD, Alzheimer’s disease.

Based on the data of the collected articles, we analyzed the relationship between different degrees of AD (mild, medium, and severe) and urine FA levels (Figure 5). The results showed that, compared with those of mild and medium AD patients, the urine FA levels of patients with severe AD increased more significantly (SMD = 1.60, 95% CI = 0.87–2.34, *p* < 0.0001; Figure 5).

### Diagnostic value of urine formaldehyde levels in Alzheimer’s disease

**FIGURE 6:**
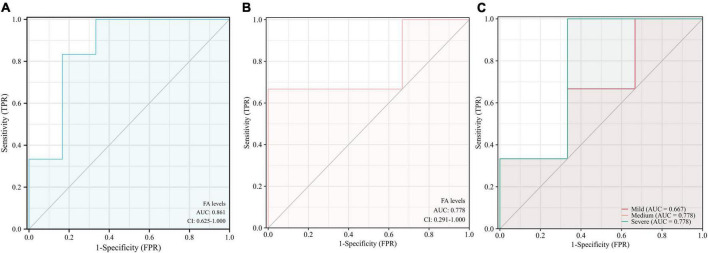
Receiver operating characteristic curve analysis of urine FA levels in patients with AD. **(A)** Detection with Fluo-HPLC. **(B)** Detection with HPLC. **(C)** Different degrees of AD (mild, medium, and severe). ROC, receiver operating characteristic curve; AD, Alzheimer’s disease; FA, formaldehyde; HPLC, high performance liquid chromatography.

The results indicate that urine FA is promising as a potential marker for the diagnosis of AD, and the diagnostic accuracy of the Fluo-HPLC assay was better than that of the traditional HPLC assay (AUC = 0.861, 95% CI: 0.625–1.00, *p* = 0.0374, Figures 6A,B). Among them, the sensitivity was 83.3%, the specificity was 83.3%, and its Youden index was calculated to be 0.667 based on its specificity and sensitivity. In addition, to explore the correlation between urine FA and AD disease grades, we have presented the diagnostic accuracy of urine FA in three degrees of AD (mild, medium, and severe) in Figure 6C. The results showed that, compared with that in mild AD patients, the diagnostic accuracy of urine FA levels in medium and severe AD patients was higher (AUC = 0.778; Figure 6C).

### Correlation analysis between urine formaldehyde levels and mini mental state examination score

**FIGURE 7:**
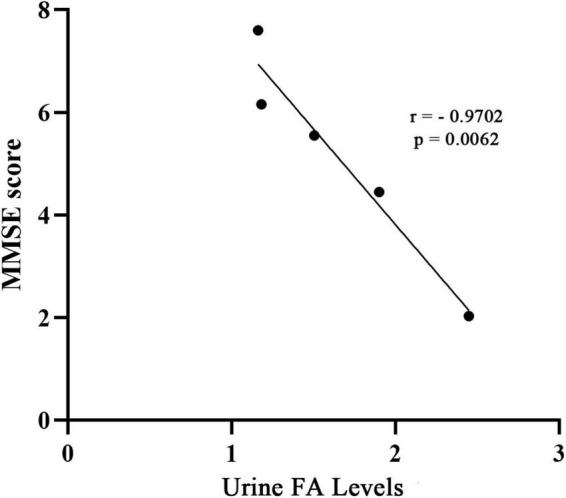
Correlation between urine FA levels and MMSE score. The concentration of FA in urine was negatively correlated with MMSE score (*r* = –0.9702, *p* = 0.0062). MMSE, mini-mental state examination; FA, formaldehyde.

As a supplement to validate the above results, and based on the data of the collected articles, this study analyzed the relationship between the urine FA levels and mini mental state examination (MMSE) scores in AD patients from the included studies. The results showed a negative correlation between the urine FA levels and mean MMSE scores in AD patients (*r* = −0.9702, *p* = 0.0062, Figure 7). This is consistent with previous conclusions that FA is negatively correlated with cognitive performance.

## Discussion

Currently, the clinical diagnosis of AD is based on the MMSE, Montreal Cognitive Assessment (MoCA) grading test combined with magnetic resonance imaging and lumbar puncture, and the detection of abnormal biomarkers (i.e., levels of Aβ and phosphorylated tau proteins) in the blood and CSF (Jack et al., 2018). Since AD is a disease that worsens with age, early diagnosis of AD is important. Given the current unsatisfactory methods for clinical diagnosis, it is urgent to find diagnostic biomarkers for AD that are rapid, convenient, and non-invasive. Due to the availability of urine and its non-invasive collection, an emphasis should be placed on the exploration of urine biomarkers for the diagnosis of AD. Therefore, the present meta-analysis was performed to investigate the association between the FA levels in urine and AD and to further assess the potential diagnostic value of urine FA levels in AD.

Aβ deposition, Tau hyperphosphorylation, and NFTs are thought to be more of a consequence than a cause of AD. Therefore, understanding what triggers protein aggregation in the brain is of great importance for research on the mechanism and treatment of AD. An increasing number of studies have elucidated the important role of FA in the pathogenesis of AD (He, 2017b). FA, a biomarker of inflammation in oxidative stress, has been associated with a variety of cognitive impairments (He, 2017a). Normally, the FA levels in the human body are maintained at 10–80 μmol/L (Szende and Tyihák, 2010). Excessive accumulation of FA can be detected in the postmortem hippocampus in AD animal models and AD patients (>0.5 mmol/L) (Tong et al., 2011, 2014, 2016). Furthermore, FA injections in the hippocampus lead to neuronal death and memory impairment (Tong et al., 2013). Previous studies also showed that FA intervention can induce classic pathological changes of AD, including Aβ deposition, Tau protein hyperphosphorylation and memory impairment *in vitro* and *in vivo* (He et al., 2016; Li and He, 2016; Jing and He, 2017; Zhai et al., 2018). Thus, the hypothesis that FA is an early event of AD was proposed.

Endogenous FA is mainly excreted through the urinary system. With increasing age, the decreased metabolic capacity of endogenous FA contributes to AD pathogenesis. The unmetabolized FA in the body can further lead to DNA damage, mitochondrial dysfunction, and neuronal apoptosis (Szende and Tyihák, 2010). Additionally, FA acts as a donor for methylation, and its excessive elevation can lead to decreased DNA methyltransferase (DNMT) activity, affecting DNA methylation levels worldwide in the brain, particularly in the hippocampus (Tong et al., 2014). This is consistent with previous studies in which postmortem examination revealed higher FA concentrations in the hippocampus of AD patients than in HCs (Tong et al., 2011). In addition, endogenous FA levels have also been found to be positively correlated with age in rodents and aged rhesus monkeys (Yang et al., 2014; Li et al., 2020). Corresponding to this, a variety of animal experiments have shown that exposure to FA can lead to shortened lifespan in flies as well as memory loss and cognitive impairment in mice and rhesus monkeys (Li and He, 2016; Zhai et al., 2018). These studies demonstrated that the results derived from our meta-analysis are acceptable, wherein urine FA levels were significantly increased in AD patients compared to those in HCs, indicating that there is a close relationship between elevated urine FA levels and AD.

Our meta-analysis found considerable heterogeneity (*I*^2^ = 95%, Figure 2). Considering possible methodological or subject age or sex differences, subgroup analyses were performed (Figure 3). Nevertheless, the heterogeneity of this meta-analysis still existed. This could have originated from a variety of aspects (the amount of specimen collected, the conditions of transportation and storage, whether the specimen was repeatedly frozen and thawed, and the use of different brands of instruments), and other factors that were not explicitly mentioned which could not be further identified by subgroup analyses. Therefore, we performed a sensitivity analysis (Figure 4A) in which we excluded individual studies one by one from the analysis, and the results showed that the high degree of heterogeneity had no effect on the conclusions of our analysis. No significant publication bias was observed (Figure 4B). This suggested that the results of our meta-analysis can be endorsed. In addition, it should be noted that while age differences did not interfere with our results, the urine FA levels have been shown to be age-related and positively correlated with age (Tong et al., 2013, 2014; Yu et al., 2014).

In this study, ROC curves were constructed according to two methods (Fluo-HPLC and HPLC) to assess the potential accuracy of urine FA levels in screening or diagnosing AD. The results of the ROC curve analysis showed that urine FA levels may be a potential diagnostic marker (AUC: 0.861, Figure 6A). The urine FA data were derived from morning fasting urine of AD patients and HCs. Although the urine FA was hardly affected by proteins (Tong et al., 2011), to obtain more reliable conclusions, we believe that future studies should: (1) Detect urine FA levels based on the same methodology. (2) Obtain more accurate raw data on urine FA levels in AD patients. (3) Detect urine FA levels in combination with their upstream and downstream related markers. (4) Describe whether patients were previously exposed to environmental FA. As additional studies are completed, we expect that the level of urine FA will become substantiated as an effective biomarker for the diagnosis of AD.

To explore the association between urine FA levels and the degrees of AD, we performed a meta-analysis of urine FA levels among patients with different degrees of AD (mild, medium, and severe). Our results were consistent with those of previous studies in which the urine FA levels increased more significantly in severe AD patients than in mild and medium AD patients (Figure 5). Afterward, we constructed ROC curves based on the urine FA levels based on the three different degrees of AD, and the results showed that the urine FA levels were more accurate for diagnosing medium and severe AD (AUC: 0.778, Figure 6C). However, the mean levels of urine FA remained higher in patients with mild and medium AD than in HCs (Figure 5). The result of the correlation analysis between urine FA levels and MMSE scores further validated the above conclusions (*r* = −0.9702, Figure 7), that is, urine FA was negatively correlated with cognitive performance in AD patients. Therefore, we hypothesize that urine FA levels may also be useful in identifying early AD. This is consistent with the conclusion that FA is one of the risk factors for the early onset of AD (Song and Wang, 2010; Tang et al., 2012; Rizak et al., 2014).

To our knowledge, this is the first study to perform a meta-analysis on the association between urine FA levels and AD. Although our results strengthen evidence for the association between elevated urine FA levels and AD, suggesting that elevated urine FA levels are a risk exposure factor for AD pathogenesis and preventing elevated FA levels may be a future strategy for the treatment of AD, our meta-analysis still had some limitations as follows: (1) We collected published case-control studies on urine FA and AD, however, few studies were available, and additional longitudinal studies with adequate sample sizes are needed to confirm our conclusions. (2) The studies we included were concentrated in the Chinese region, and there were no data from other countries to support our conclusions. (3) Due to the significant differences in the methodologies for detecting urine FA levels, the mean urine FA levels of the included studies were significantly different. (4) The included studies did not specify whether AD patients were previously exposed to environmental FA. In summary, our meta-analysis suggests that urine FA levels are considerably higher in AD patients than in HCs. FA levels may be a potential non-invasive biomarker to aid in the diagnosis of AD. However, due to the limitations and heterogeneity of the study, further experiments are warranted to verify our conclusions.

## Conclusion

Our meta-analysis elucidates the close relationship between elevated urine FA levels and AD. However, there are few studies on FA and AD, and there is no available data from countries other than China to support our conclusions. Our study suggests that the prevention of elevated urine FA levels may be a novel strategy for the treatment of AD. While our results indicate the potential accuracy of urine FA for the diagnosis of AD, additional longitudinal studies that are well-designed and case-control are needed to further examine the relationship between FA and AD pathogenesis.

## Data availability statement

The original contributions presented in this study are included in the article/supplementary material, further inquiries can be directed to the corresponding authors.

## Author contributions

FC and NW designed and carried out the study. FC and XH wrote the manuscript with other authors’ input. XH and RH provided scientific discussion and revised the manuscript. JS advised on data presentation and provided financial support. XT and YQ participated in analyzing and interpretation of the results. All authors contributed to the article and approved the submitted version.
